# Retraction: Shikonin suppresses proliferation and induces apoptosis in endometrioid endometrial cancer cells via modulating miR-106b/PTEN/AKT/mTOR signaling pathway

**DOI:** 10.1042/BSR-2017-1546_RET

**Published:** 2024-02-28

**Keywords:** ant-cancer, endometrioid endometrial cancer, microRNAs, miR-106b/PTEN/AKT/mTOR signaling pathway, Shikonin

This article is being retracted from *Bioscience Reports* at the request of the Editor-in-Chief and the Editorial Board following receipt of a notification from a reader, alerting the Editorial Board to images in this article that are published elsewhere including:

- Figure 7A Ishikawa B-actin, with Figure 4E WM266-4 B-actin from Du et al 2017 (doi: 10.1186/s40659-017-0141-8) and Figure 2C SW620 B-actin Blank from Sun et al 2016 (doi: 10.1042/bsr20160048)
- Figure 7C HEC-1A ATK, with Figure 2C HT29 BCL-2 from Sun et al 2016 (doi: 10.1042/bsr20160048)

The authors have been contacted with regards to the duplications but did not confirm their agreement to the retraction. Given the extent of the issues raised, the Editorial Board stand by the decision to retract the article.

